# E-scooter-related orthopedic injuries and the treatments applied: are these scooters a new means of transportation or a new source of trauma?

**DOI:** 10.1186/s12873-023-00873-z

**Published:** 2023-09-19

**Authors:** Servet İğrek, İbrahim Ulusoy

**Affiliations:** Department of Orthopaedics and Traumatology, Selahaddin Eyyubi State Hospital, Diyarbakır, Turkey

**Keywords:** Electric scooter, Injury, Orthopaedic, Trauma, Fracture, E-scooter

## Abstract

**Introduction:**

E-scooters have become increasingly popular in Turkey due to easy accessibility. In parallel with this, the number of e-scooter-related injuries has increased gradually. The aim of this study was to determine the types of e-scooter-related orthopedic injuries, to evaluate hospitalization and surgical treatments, and to investigate the loss of work of patients and the burden incurred by the healthcare system.

**Materials and methods:**

This retrospective multicentre study included all orthopaedic referrals, who were admitted to two major trauma departments in Diyarbakır from January 2022 to July 2022. Patient data were analyzed, including demographic characteristics, injury pattern, types of injury and subsequent treatment.

**Results:**

In a total of 62 patients, 105 orthopaedic injuries were identified, comprising 72.5% males and 27.5% females, with a median age of 34.21 years. Fifty-six (90.3%) patients were riders, and six were pedestrians. All associated e-scooters were rented. There were 44 fractures (41.9% of the total recorded injuries) including 8 (12.9%) open fractures. Surgery was required by 32 patients (51.6%) and 35 (56.4%) required hospital admission leading to hospitalization of 3.7 days on average. The average job loss of working patients after injury was determined as 2.4 months. Helmet use was detected in 6.4% of the e-scooter users, but no other protective equipment was detected in any of the patients. Furthermore, 19,3% of the patients had a blood alcohol level of > 10 mg/dl.

**Conclusions:**

The injuries that may result from an e-scooter accident can have long-term hospitalization and long-term job loss in the community. This imposes a significant financial burden on the national healthcare system and adversely affects public health. There is a need for precautions to be implemented such as infrastructure organisation, increased awareness of motor vehicle riders and e-scooter riders, and increased enforcement of rule compliance including not using e scooters after alcohol consumption and the use of protective equipment.

## Introduction

The use of electric scooters (e-scooters), which has been increasing in frequency especially in Europe, America and Asia since 2017, has become quite common all over the world since the COVID-19 pandemic [1–4]. With the development of the e-scooter rental system in Turkey at the end of 2019, they started to be used frequently, especially in big cities [5]. The fact that they can be rented via smart phones and are easily accessible in public places has increased the popularity of e-scoooter use among the public. The most important advantages of e-scooters are that they reduce traffic density and carbon monoxide emissions in big cities [6], e-scooters pose less parking hassle than conventionally driven automobiles, they provide a cheap form of transportation and allow individual transportation in terms of preventing infectious diseases. However, in addition to all these beneficial features, with the introduction of e-scooter use into daily life, e-scooter-related injury rates have increased significantly all over the world [1–4, 7, 8].

Wearing protective equipment and obtaining a driver’s license is not obligatory when using e-scooters in Turkey. It is not prohibited to use an e-scooter while intoxicated, but it is prohibited to use them under the age of 15 years, to use them on pavements, to have more than one person riding the scooter, and to exceed 20 km/h. In addition, due to the increasing number of accidents, riders are required to wear reflective vests at night. All these measures have not led to a decrease in accidents, but in contrast, with increased frequency of use the number of accidents has also increased. Despite the frequent reports of accidents in the Turkish media, the literature on the subject is quite limited [5]. The hypothesis of this study was that e scooter-related injuries are high-energy injuries, the use of protective equipment is low, and the injuries place a heavy burden on the healthcare system. Therefore, the aim of the study was to determine the injury patterns after e-scooter accidents, to evaluate hospitalization and surgical treatments, and to investigate the loss of work of patients and the burden incurred by the healthcare system.

## Materials and methods

**Fig. 1:**
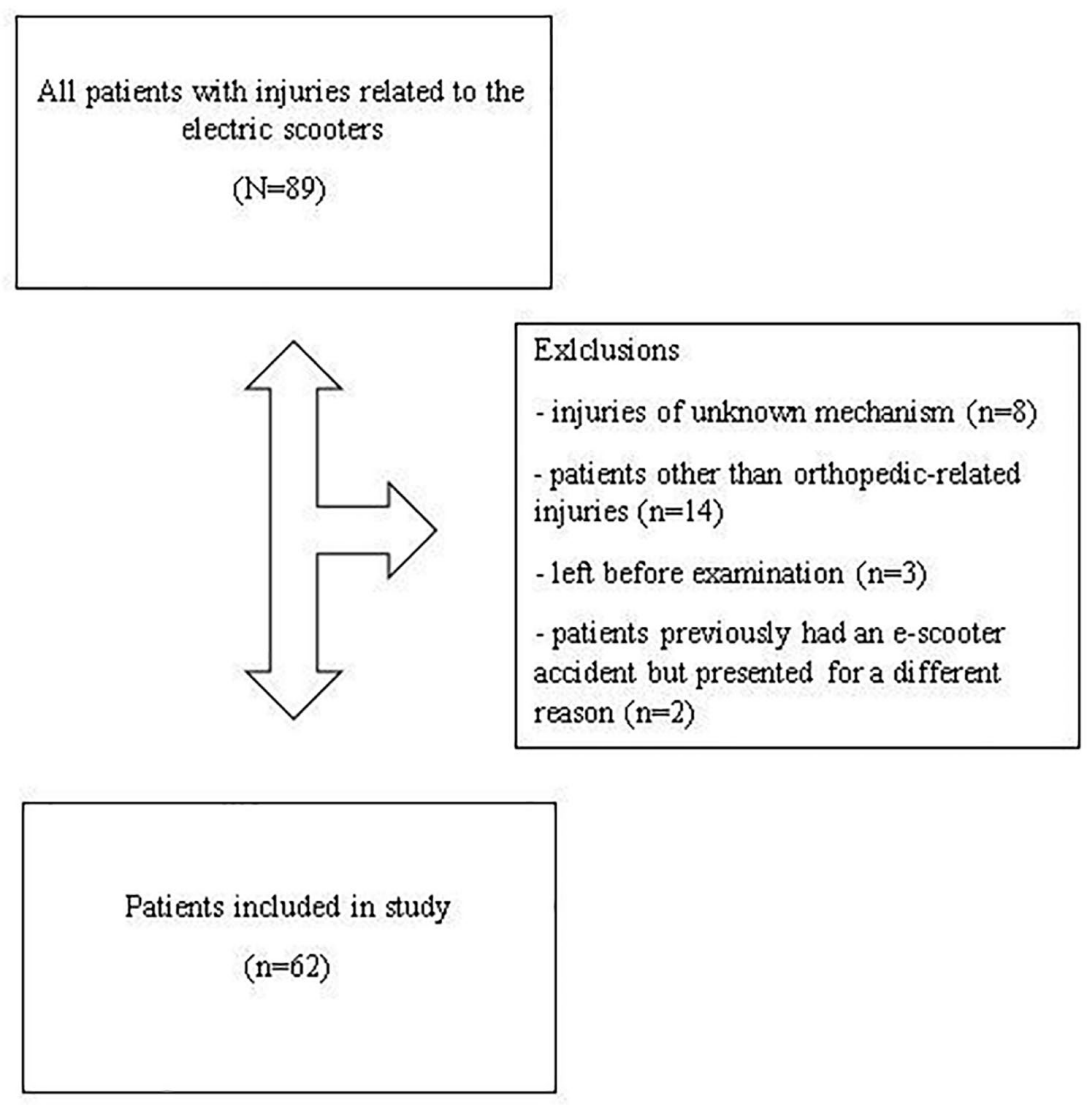
Flow chart

A retrospective analysis was made of all patients who presented at Diyarbakır Gazi Yaşargil Training and Research Hospital (level 3 trauma centre) and Diyarbakır Selahaddin Eyyubi State Hospital (level 2 trauma centre) emergency service with orthopedic injury after an e-scooter accident between January and July 2022 and were operated on in the Orthopedics and Traumatology Department. Patient data were obtained from the hospital database. Approval for the study was granted by the Institutional Review Board. The study was conducted in accordance with the principles of the Helsinki Declaration. All patients who presented at the Emergency Department, either as a rider or pedestrian, with an e-scooter-related orthopedic injury were included in the study. Patients who were excluded from the study if the mechanism of injury was not clear or if they had previously had an e-scooter accident but presented for a different reason. Patients left before examination or patients other than orthopaedic related injuries also excluded from the study. See flow chart in Fig. 1.

A record was made for each patient of the injury pattern, time of admission, mechanism of injury, treatment applied, duration of job loss due to medical report obtained after treatment, Glasgow Coma Scale (GCS) score at the time of admission, illegal substance use, and drug or alcohol use. The age and gender of the patients and whether they had been wearing a helmet or protective equipment such as reflective vests, knee and elbow pads at the time of the accident were determined. Riders were separated into 3 age groups as < 15 years, 15–40 years, and > 40 years. The place where the accident occurred was classified as a pavement, street, or cycle path. Patients were grouped as riders and non-riders. All the injuries were classified according to the Injury Severity Score (ISS) [9]. An ISS score of 1–8 is defined as minor trauma, 9–15 as moderate trauma and greater than 15 as major or polytrauma. The development of mortality during or after admission to the emergency department, those who were admitted to a ward or the intensive care unit, and those who were discharged from the emergency room were determined. Injury sites were grouped as upper extremity, lower extremity, spine, and pelvis, and injury patterns as fracture, dislocations and isolated soft tissue injury. Fractures were categorised in 2 groups as open and closed.

### Statistical analysis

Data obtained in the study were analyzed statistically using NCSS (Number Cruncher Statistical System) 2007 software (Kaysville, Utah, USA). Descriptive analyses were performed. Absolute numbers and percentages for variables were reported. Comparisons between independent groups of continuous variables were performed using the Independent Samples t-test and the Chi-square test. The level of statistical significance was set at p < 0.05.

## Results

A total of 62 patients with orthopedic injuries after an e-scooter accident presented at the emergency services of two hospitals in a 7-month period. Since the rental program in Diyarbakır started in January 2022, no patient presented to the emergency department due to an e-scooter accident before this date. The patients comprised 45 (72.5%) males and 17 (27.5%) females, with a mean age of 34.21 years (min 9; max 58). The age group distributions showed that 16 (25.8%) patients were under 15 years old, 30 (48.3%) were aged 15–40 years, and 10 (16.1%) were over 40 years. It was determined that 56 (90.3%) patients were riders, 6 (9.7%) were non-riders, and 12 (19.3%) were riding on the e-scooter with two people. The injury characteristics and patient data are shown in Table 1.

**Table 1:** Patient and accident characteristics for orthopaedic referrals associated with e-scooters

	n (%)
Age in years(mean[range])	34.21 [9–58]
Age < 15 years	16 (25.8%)
15 ≤ Age ≤ 40 years	30 (48.3%)
40 < Age years	10 (16.1%)
Male gender	45 (72.5%)
Rider	56 (90.3%)
Non-rider	6 (9.7%)
Mechanism of injury	
Fall	42 (75%)
Collision with a moving motor vehicle	4 (7.1%)
Collision with a bicycle	2 (3.55%)
Collision with another e-scooter	2 (3.55%)
Collision with a stationary object or person	6 (10.8%)
Blood alcohol level > 10 mg/dl	12 (19.3%)
Helmet use	4 (6.4%)
Other protective equipment use	-
Place of injury	
Cycle path	28 (45.1%)
Road	22 (35.4%)
Pavement	12 (19.5%)
Time of injury	
5 pm to 8 am on weekdays and at weekend	41 (66.1%)
8 am to 5 pm on weekdays	21 (33.9%)
Type of treatment	
Non-operative management	30 (38.5%)
Operative management	32 (61.5%)
Mean hospital stay	3.7 days
GCS score < 8	1(1.6%)
Average job loss of patients	2.4 months
Discharged from ED	27 (43.6%)
Admitted to ward	34 (54.8%)
Intensive care unit	1(1.6%)
Minor trauma according to ISS	38 (61.4%)
Moderate trauma according to ISS	23 (37%)
Major trauma according to ISS	1(1.6%)

ED: Emergency Department ISS: Injury Severty Score

The most common mechanism of injury in riders was falling, which was seen in 42 (75%) patients. Collision with a moving motor vehicle was reported in 4 (7.1%) patients, with a bicycle in 2 (3.55%) patients, with another e-scooter in 2 (3.55%) patients, and with a stationary object or person in 6 (10.8%) patients. The blood alcohol level was determined to be > 10 mg/dl in 12 (19.3%) patients and all intoxicated patients were riders. Helmets were worn by 4 (6.4%) patients and no other protective equipment was being used by any of the patients. The accidents occurred on a cycle path in 28 (45.1%) cases, on a road in 22 (35.4%) cases, and on a pavement in 12 (19.5%) cases. The majority (66.1%) of accidents occurred either on the weekend or at nighttime between 5 pm and 8 am on weekdays (Table 1).

In the total of 62 patients, 105 injuries were detected, with more than one injury observed in 32 (51.6%) patients. There were determined to be 44 (41.9%) fractures of which eight were open. Shoulder dislocations were detected in 8 patients and elbow dislocations in 2 patients. The fractures were localized to 23 (52.2%) upper extremity, 16 (36.3%) lower extremity, 3 (6.8%) pelvis and 2 (4.5%) spine fractures (Table 2). Surgical treatment was applied to 32 (51.6%) patients (Table 3). The mean hospital stay was 3.7 days. The GCS of 1 patient was < 8 after coming to the Emergency Department. According to the ISS, minor trauma was detected in 38 (61.4%) patients, moderate in 23 (37%) patients, and major trauma in 1 (1.6%) patient, and the mean ISS value was 4.2. While the mean ISS was 11.2 in the patient group over 40 years of age, it was 3.5 in the group of patients under 40 years of age. The average job loss of working patients after injury was determined as 2.4 months. While 27 (43.6%) patients were discharged from the emergency department, 34 (54.8%) patients were hospitalized and 1 (1.6%) patient with polytrauma died after 2 days of intensive care.

**Table 2:** Anatomical distribution of fractures and injury characteristics

	n (%)
Total number of injuries	105
Fracture	44 (41.9%)
Open fractures	8 (18.2%)
Closed fractures	36 (81.8%)
Dislocations (no fracture)	10 (9.5%)
Shoulder	8 (80%)
Elbow	2 (20%)
Isolated soft tissue injuries	51(48.6%)
Elbow	17 (33.3%)
Knee	12 (23.5%)
Ankle	9 (17.6%)
Wrist	13 (25.6%)
Anatomical location of fractures	
Upper extremity fracture	23 (52.2%)
Clavicle	2
Humerus	3
Radius	8 (1 of which were elbow fracture dislocation)
Ulna	5
Hand	5
Lower extremity fracture	16 (36.3%)
Femoral shaft	1
Tibial plateau	2
Patella	2
Tibial shaft and fibula	2
Ankle	3
Foot	6
Pelvis	3 (6.8%)
Iliac wing fracture	2
Pubic ramus fracture	1
Spine	2 (4.5%)

**Table 3:** Types of surgeries

Open reduction/internal fixation	18
External fixation	1
Manipulation under anaesthesia/closed reduction	2
Intramedullary device	2
Arthroscopic shoulder surgery	1
Percutaneous pinning	5
Isolated soft tissue repair	3

The time of the injury of the patients who were found to be intoxicated when the accident occurred was determined at a statistically higher rate out of working hours and at weekends (p = 0.0310). Fractures were found in 10 (83.3%) of the intoxicated patients and 9 (75%) of these patients were treated surgically. A statistically significantly higher fracture rate was determined in patients who were intoxicated at the time of the accident, and the number of patients who underwent surgical treatment in this group was statistically higher than that of the patients who had not drunk alcohol (p = 0.0214; p = 0.0312). In the analyses performed, it was observed that the ISS was higher in patients over 40 years of age compared to the younger group (p = 0.0432).

## Discussion

**Fig. 2:**
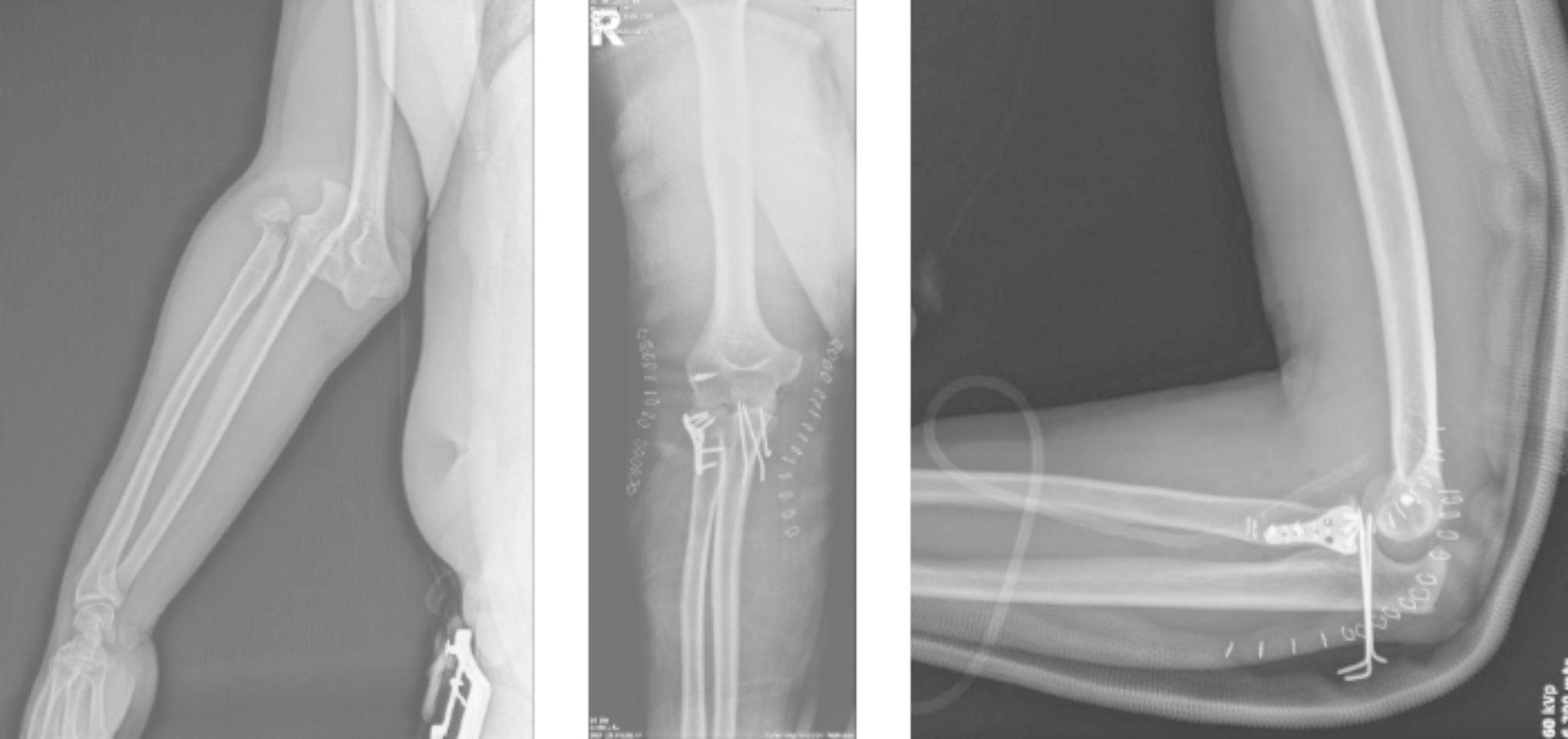
A 23-year-old male patient with elbow fracture dislocation (terrible triad) after falling from e-scooter. (A) Preoperative antero-posterior radiography, (B) Postoperative antero-posterior radiography, (C) Postoperative lateral radiography

**Fig. 3:**
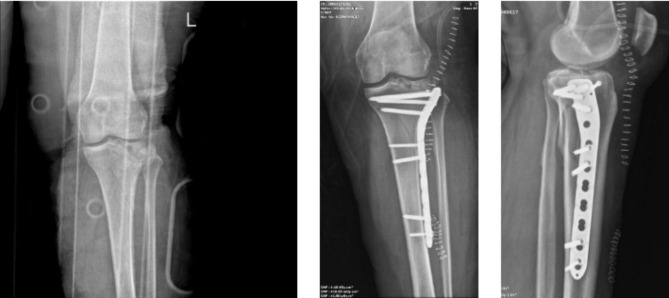
A 45-year-old male patient with tibial plateau fracture after falling from e-sccoter. (A) Preoperative antero-posterior radiography, (B) Postoperative antero-posterior radiography, (C) Postoperative lateral radiography

To the best of our knowledge, this study is the first to have evaluated orthopedics-related injuries after e-scooter accidents in Turkey. In particular, people’s avoidance of public transport during the COVID-19 pandemic, the transition to a shared e-scooter system in many big cities, and cheap and easy use have made the use of e-scooters widespread among the public. However, the number of accidents has increased rapidly. As seen in this study, various traumas that affect human life, including death, may occur after using e scooters. From the study results, it was observed that 32 (61.5%) patients were operated on due to fractures and dislocations after the accident (Figs. 2 and 3). In a study by Cruz et al., surgery was performed on 30.1% of the patients and it was shown that the use of e-scooters caused high-energy injuries such as femoral neck fractures, open lower and upper extremity fractures, and fracture-dislocations [10]. In another study, major musculoskeletal injuries were observed at the rate of 36% after e-scooter accidents [11]. Mayhew et al. reported that orthopedic injuries after e-scooter accidents were the most common pathology at the rate of 20.6% [1]. Similar to the current study, previous studies have shown that the most common fractures after an e-scooter accident are upper extremity fractures [8, 10, 12].

In the current study, mortality occurred in 1 (1.6%) e-scooter rider while the patient was in the intensive care unit in the hospital after being hit by a motor vehicle. In addition to abdominal injury, the patient also had pelvis and femoral shaft fractures. After the first intervention, in which an external fixator was applied to the pelvis and femur, the patient died due to ARDS. Mayhev et al. have also reported deaths after e-scooter accidents [1]. In the current study, 34 patients (54.8%) were hospitalized on wards and the mean hospitalization period was 3.7 days. Genc et al. reported that after a motor vehicle collision with an e-scooter, one patient was admitted to the intensive care unit due to cranial and thoracic injuries, in addition to femur and humerus shaft fractures [5]. Trivedi et al. Reported 6.0% ward hospitalization and 0.8% intensive care admission [11]. Cruz et al. reported a hospitalization rate of 34.9% with an average length of stay of 4.9 days [9]. At the same time, in the current study, the average job loss after the accident was found to be 2.4 months. All of this shows the high costs that e-scooter accidents bring to the healthcare system and the negative effects on public health.

Of the e-scooter riders in the current study, 12 (19.3%) were found to be under the influence of alcohol. It has been previously observed that the rate of fractures increases in accidents after alcohol consumption, and especially the rate of fractures that require surgery. This can be explained by the difficulty in maintaining balance and loss of reflexes after alcohol intake. Alcohol-related accidents have been observed to occur more after 5 pm on weekdays and at weekends. In a previous study, alcohol was detected in 4% of patients after an accident, and similar to the current study, the risk of major trauma was seen to be increased in this patient group. All of these patients had the accident after 8 pm [4]. In another study, alcohol was detected in 7.2% of riders after an e-scooter accident, and it was stated that the introduction of a licence for e-scooters would reduce the rate of alcohol-related accidents [10]. In another study, the patients involved in e-scooter accidents were determined to be mostly young adults under the influence of alcohol and the injuries were usually facial trauma [13]. In Turkey, there is no regulation on the use of e-scooters and alcohol. During routine police checks, e-scooter riders are not checked, so there is no deterrant to riding an e-scooter after alcohol consumption. In the current study, it appears that 66.1% of accidents occurred either on a weekend or between 5pm and 8am on weekdays. In another study, it was shown that 55.4% of the accidents occurred between 5 pm and 8 am [10], and in yet another, that 58.3% of the accidents occurred at night time [4]. These data suggest that the e-scooter is mostly used by the public as a means of entertainment as well as being a means of transportation. Considering that alcohol consumption also increases at night, banning the use of e-scooters after a certain hour at night, prohibiting alcohol consumption, and applying stricter controls could reduce accident rates.

The current study results showed that 28 (45.1%) of the accidents occurred on a cycle path, 22 (35.4%) on a road, and 12 (19.5%) on a pavement. Cruz et al. reported that accidents occurred most frequently on roads (65.1%) and pavements (32.9%). The use of e-scooters on pavements is prohibited in Turkey and there is no specific area for e-scooter use. Riders mostly use the cycle paths if available, and if not, they prefer pavements and roads. As seen in the current study, accidents occur as a result of collisions with bicycles when using cycle paths, collisions with pedestrians when using pavements, and collisions with motor vehicles on roads. This demonstrates that there is insuficient infrastructure for e-scooters, which have rapidly come into widespread use. Cycle lanes were introduced for built for bicycles, which were already in use and with the recent use of these lanes by e-scooters, the traffic density has increased and accidents have become inevitable. E-scooters are prohibited on pavements but continue to be used despite this ban, so they cause accidents by hitting people especially on busy pavements. Accidents that occur on roads can be more severe due to collisions with motor vehicles [14]. In the current study, the patient who died had an accident by colliding with a motor vehicle on the road. With various infrastructure re-arrangements and raising the awareness of riders, e-scooter accidents on the roads could be prevented and the use of e-scooters could become safer.

Although it is forbidden to use e-scooters under the age of 15 years in Turkey, the current study results showed that 25.8% of the riders were under the age of 15 years. In a study by Genc et al., 8.5% of the patients were under the age of 18 years [5], and Cruz et al. reported that 16.9% of patients were aged < 16 years. It has been stated that injuries in this age group can be prevented by enforcing the age-limited e-scooter license [10]. One of the important results of the current study was that the ISS was higher in patients over 40 years of age than in younger patients. Moftakhar et al. also showed that the ISS was higher in elderly patients than in younger patients [4]. This demonstrates that the high-risk group is not only patients under the age of 15 years, but also that riders in the older age group should be more careful when using e-scooters.

The important limitations of this study were the retrospective design, the short time period and that the focus was only on orthopedic injuries. Patients who did not have orthopedic injuries after an e-scooter accident and who presented at the emergency room with different injuries were not evaluated. This prevented the evaluation of scooter accidents in their entirety. Nevertheless, strong aspects of the study were that it was a multicentre study in a large urban centre and it is the first study to have reported e-scooter-related orthopedic injuries in Turkey. Further studies are required to fully understand the long-term impact of e-scooter-related injuries on the patient and the economic burden on the healthcare system.

## Conclusions

The use of e-scooters is becoming more and more common throughout the world and in Turkey. The most important advantages of this form of travel are that it provides cheap and easy transportation and, is environmentally friendly. However, the use of e-scooters can cause injuries with long-term hospitalizations and long-term job loss in the community. Therefore, e-scooters impose a significant financial burden on the national healthcare system, and adversely affect public health. The regulations implemented by the government related to e-scooters are not sufficient to reduce the accident rate. The establishment of better infrastructure, with the construction of new roads if necessary, raising awareness of e-scooter riders, motor vehicle riders and pedestrians, increased enforcement of compliance with the rules, not using e-scooters after alcohol consumption and the use of protective equipment pose as potential solutions to mitigating and minimizing the occurrence of e-scooter related orthopaedic traumatic injury.

## Data Availability

All data generated or analysed during this study are included in this published article.
